# Promoter Sequence Determines the Relationship between Expression Level and Noise

**DOI:** 10.1371/journal.pbio.1001528

**Published:** 2013-04-02

**Authors:** Lucas B. Carey, David van Dijk, Peter M. A. Sloot, Jaap A. Kaandorp, Eran Segal

**Affiliations:** 1 Department of Computer Science and Applied Mathematics, Weizmann Institute of Science, Rehovot, Israel; 2 School of Computer Engineering, Nanyang Technological University, Singapore; 3 Computational Science, University of Amsterdam, Amsterdam, The Netherlands; Albert Einstein College of Medicine, United States of America

## Abstract

The ability of cells to accurately control gene expression levels in response to extracellular cues is limited by the inherently stochastic nature of transcriptional regulation. A change in transcription factor (TF) activity results in changes in the expression of its targets, but the way in which cell-to-cell variability in expression (noise) changes as a function of TF activity, and whether targets of the same TF behave similarly, is not known. Here, we measure expression and noise as a function of TF activity for 16 native targets of the transcription factor Zap1 that are regulated by it through diverse mechanisms. For most activated and repressed Zap1 targets, noise decreases as expression increases. Kinetic modeling suggests that this is due to two distinct Zap1-mediated mechanisms that both change the frequency of transcriptional bursts. Notably, we found that another mechanism of repression by Zap1, which is encoded in the promoter DNA, likely decreases the size of transcriptional bursts, producing a unique transcriptional state characterized by low expression and low noise. In addition, we find that further reduction in noise is achieved when a single TF both activates and represses a single target gene. Our results suggest a global principle whereby at low TF concentrations, the dominant source of differences in expression between promoters stems from differences in burst frequency, whereas at high TF concentrations differences in burst size dominate. Taken together, we show that the precise amount by which noise changes with expression is specific to the regulatory mechanism of transcription and translation that acts at each gene.

## Abstract 2

A single transcription factor can activate or repress expression by three different mechanisms: one that increases cell-to-cell variability in target gene expression (noise) and two that decrease noise.

## Author Summary

In response to environmental changes, cells regulate the activity of transcription factors (TFs), which in turn change the expression of dozens of downstream target genes by binding to their promoters. The response of each target gene is determined by the interplay between TF concentration and the context in which TF binding sites occur in each target promoter. To examine the relationship between promoter sequence, mechanism of regulation, and response to TF activity, we measured expression of 16 target genes of a single TF in response to changes in TF concentration in single cells. We found that different native promoters that are all targets of the same TF exhibit diverse responses to changing TF levels in terms of both gene expression level and cell-to-cell variability (noise) in expression. Using computational modeling and mutations of specific promoter elements, we show that the molecular mechanisms of regulation can be inferred by measuring how noise changes with expression. These results show that a single TF can regulate transcription through multiple mechanisms, resulting in similar changes in mean expression but vastly different changes in cell-to-cell variability.

## Introduction

The cellular response to environmental changes is mediated through activation of TFs and subsequent coordinated activation and repression of dozens of target genes. However, gene expression is noisy [1], and this limits the precision with which cells can regulate protein levels. Genome-wide, noise (σ^2^/μ^2^, variance/mean^2^) decreases as expression increases [2]–[4]. Along this global trend, individual genes with the same average expression in the population differ in their amount of noise. The level of noise for each gene is related to its function and is determined by the mechanisms of regulation [5]. However, the precise mechanisms by which control of noise is accomplished for native genes are not known.

Two quantities that describe the dynamics of gene expression, and have been related to the distribution of protein abundances, are burst size and burst frequency. Burst frequency is determined by the rate at which the promoter switches from an inactive to an active transcriptional state due to transcription factor (TF) binding and subsequent PolII recruitment (promoter on-switching). Burst size is the number of proteins produced during each promoter on-event [6]–[8]. Native genes differ in the relative contribution of burst frequency and size to expression [4],[9],[10], suggesting that evolution can tune both parameters in order to reach an optimal level of expression and noise for each gene [11].

When an increase in gene expression is caused by an increase in the rate of promoter on-switching (burst frequency), noise (σ^2^/μ^2^) decreases monotonically with expression [12]. In contrast, an increase in burst size (due to a decrease in promoter off-switching rate or an increase in the transcription or translation rate) results in an increase in expression and in noise strength (σ^2^/μ), and no change in noise [12]. Mutations in the TATA box in yeast [13] and the ribosome binding site in *B. subtilis* [14] both affect noise strength, but not noise. The former is thought to be involved in transcription re-initiation [15], thus extending the time of each active state of the promoter, while the latter affects the number of proteins produced from each mRNA molecule. These observations strengthen the claim that changes in mean expression but not noise stem from molecular mechanisms that affect the number of proteins produced during each transcriptional event, but not the frequency of such events. Taken together, these data support a model of gene expression in which changes in promoter dynamics, such as changes in on-switching rates and transcription and translation rates, can be deduced by measuring how noise changes with expression [4],[7],[8].

Since most genes are regulated through multiple mechanisms, each of which can affect burst size and burst frequently differently, different genes should exhibit different relationships between mean expression and noise. However, measurements of a set of seven different promoters in *E. coli* all showed similar changes in expression and noise throughout induction [16]. Gene regulation in eukaryotes is more complex, and we hypothesized that burst frequency and burst size would be differentially regulated for each gene and, as a consequence, that the relationship between noise and expression would be different for different genes.

To characterize the relationship between mean expression and noise for native promoters in response to environmentally stimulated changes in TF activity, we generated a set of 16 strains in which distinct promoters are fused upstream of a yellow fluorescent protein reporter (YFP). In each strain, we extracted a different Zap1 binding-site containing promoter from its native locus, integrated it into the *his3* locus, and measured its expression and noise at 12 different zinc concentrations (induction levels). Decreasing zinc concentration increases the activity and expression of Zap1 and changes the expression of Zap1 target promoters [17]. The resulting Zap1 dose-response curves of these targets show activation, repression, and a combination of activation and repression, consistent with previous observations [17].

We found that for Zap1-activated targets, an increase in Zap1 causes an increase in expression and a decrease in noise. Similarly, Zap1-repressed targets exhibit the same relationship between expression and noise, whereby an increase in Zap1 causes a decrease in expression and an increase in noise. Despite this general trend that has previously been reported [2]–[4], we found that the slope of noise versus expression is unique for each promoter, showing that noise is not determined by expression level alone. The most notable exception to expression determined noise is the *ZRT2* promoter, which is both activated and repressed by Zap1 [18], in which we found a different and novel relationship between mean expression level and the distribution of expression. Repression of *ZRT2* by Zap1 results in a decrease in both expression and noise, leading to a transcriptional state of low expression and low noise that is unique among the 16 tested promoters. This behavior is predicted by a kinetic model in which repression is due to a secondary binding event near the TATA that causes a decrease in transcription rate (burst size), thereby preventing the typical increase in noise that accompanies repression due to a reduction in burst frequency. These results suggest that the relationship between noise and expression is unique to each promoter and is determined by the regulatory mechanism encoded in the promoter DNA sequence and not by mean expression level alone.

We hypothesized that further noise reduction will occur when activation and repression are performed by the same TF. Using a model of noise that takes into account the sensitivity to TF level fluctuations and an experiment in which we decouple activator from repressor, we find strong evidence supporting our hypothesis that coupling between activator and repressor is a mechanism for noise reduction.

Finally, analysis of the data from all measured Zap1 targets brings forward a global principle of regulation in which the major source of differences in expression between promoters changes with induction. Our results strongly support a model in which at low Zap1 activity, differences in expression between Zap1 targets are due to variability in the frequency of transcriptional bursts, while at high Zap1 activity, differences are due to variability in the number of proteins produced during each transcriptional burst. This model suggests that such behavior is a general property of transcriptional regulation.

## Results

### Each Target of a Single TF Exhibits a Unique Gene-Specific Scaling of Expression and Noise in Response to Changes in TF Activity

**Figure 1:**
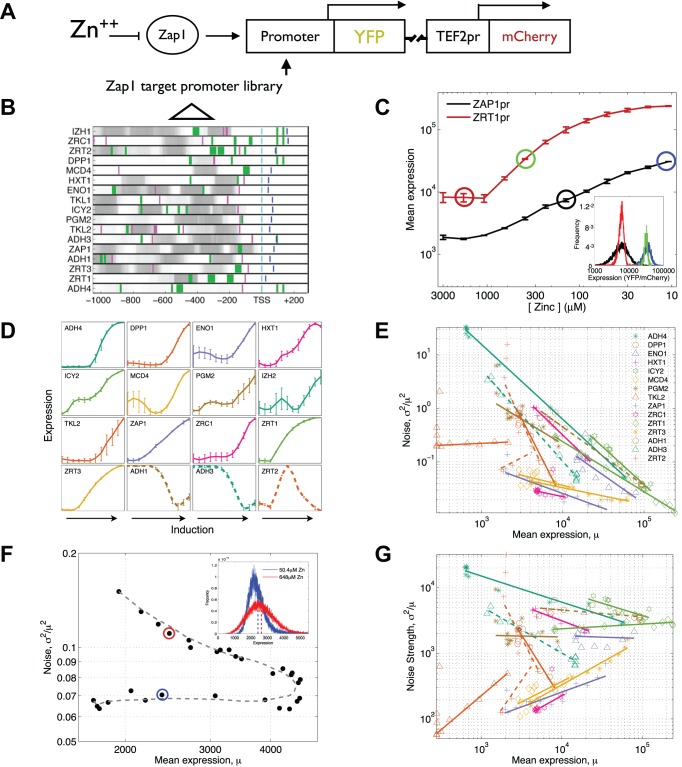
Measuring mean promoter activity and cell-to-cell variability for a library of Zap1 target promoters. (*A*) The transcription factor Zap1 is induced by decreasing the concentration of zinc in the growth medium. A schematic of the site of chromosomal integration for measuring promoter-driven expression is shown. Each yeast strain has a single promoter inserted upstream of the YFP coding sequence. At the same locus a constitutively expressed mCherry is also integrated, which is used to normalize the YFP signal and correct for extrinsic cell-to-cell variability. (B) For each Zap1 target promoter the predicted locations of the major architectural features are shown. Promoters are aligned by the transcription start site (TSS) (cyan). PSSMs for the TATA box (purple) [36] and Zap1 (green) [17] were used to predict binding sites for TBP and Zap1, respectively. The width of the green bars is proportional to the predicted affinity of each Zap1 binding site. Darker shades of grey show regions with higher predicted nucleosome occupancy. Blue lines show translation start sites. (C) Zap1 activates its own transcription, in addition to other target promoters, such as Zrt1. Shown is the measured expression (the ratio between YFP and mCherry fluorescence) of the *ZAP1* promoter and the activated target ZRT1, graphed against the concentration of zinc added to the growth media. The inset shows the single-cell distribution of measured fluorescence intensities for ZAP1 and *ZRT1* at two zinc levels obtained from flow-cytometry. (D) Measured promoter-driven expression (quantified as the ratio between YFP and mCherry fluorescence) throughout the Zap1 induction is shown for each measured promoter. Each point shows the average of at least four biological replicates. (E and F) Noise and noise strength graphed against mean expression for each promoter that changes expression by more than 2-fold. The line η^2^ = cμ^k^ was fit (solid lines) to the induction data per promoter, showing that different promoters show different scalings of noise and mean expression. (G) The measured expression distribution for the *ZRT2* promoter at two different zinc induction levels (50.4 µM and 648 µM zinc, blue and red points in E and G) with the same mean expression level but different distributions. The mean expression level for each distribution is marked with a dashed line.

To study how expression of different native promoters is regulated by environmental-induced changes in TF activity, we measured promoter-driven expression in single cells for 16 targets of the TF Zap1 in response to changes in extracellular zinc. To do this we used an experimental system that we previously developed in which a promoter of interest drives YFP expression from the genomic *his3* locus (Figure 1A) [19]. We generated a set of 16 promoter-YFP fusion strains and used flow-cytometry to perform quantitative single-cell measurements of promoter-driven expression at 12 induction points (Figure 1C). These promoters (Figure 1B, Table S1) have diverse activation curves (Figure 1D, Figure S1) and, while the response of each promoter correlates with the predicted Zap1 occupancy along the promoter (Figure S2), the diversity of responses suggests that the way in which Zap1 alters expression is different for different promoters. In addition, we examined the changes in noise and noise strength along the induction curves (Figure 1E,G). For most activated (11/13) and repressed (2/3) promoters, noise decreases as expression increases (Figure 1E, average Pearson correlation for all promoter of −0.73, Figure S3A), consistent with observed genome-wide trends [2]–[4]. In contrast, noise strength changes less consistently across Zap1 targets (Figure 1G, average Pearson of −0.09, Figure S3B). Surprisingly, not only do different promoters exhibit different amounts of noise at the same level of expression (Figure 1C), but also the way in which noise and noise strength change with expression is unique to each promoter (Figure 1E,G, Figure S4). Interestingly, a single promoter (*ZRT2*) that is both activated and repressed by Zap1 (Figure 1D, lower right) [18] shows very different amounts of noise at the same mean expression (Figure 1F). Because different molecular mechanisms of gene regulation can lead to the same change in mean expression but different changes in noise [20], these results suggest that the precise molecular mechanism by which a change in Zap1 activity causes a change in expression may be different at each promoter.

### A Kinetic Model of Promoter Switching Replicates the Experimentally Observed Changes in Expression and Noise for the *ZRT1* Promoter

**Figure 2:**
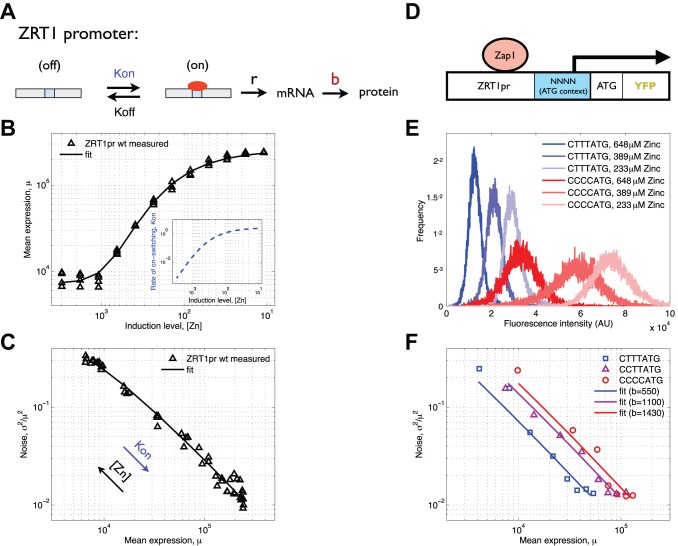
Measured and modeled gene expression of ZRT1. (A) ZRT1 expression is modeled with a kinetic scheme in which the promoter switches between a transcriptionally active (on) and inactive (off) state as a result of Zap1 (red oval) binding and unbinding. (B) Experimentally measured *ZRT1* promoter-driven expression changes as a function of zinc concentration (triangles). The kinetic model in (A) fits (line) the data (triangles) when zinc is assumed to change *Kon* (inset). (C) Noise graphed as a function of expression for the data and model from (B). (D) A schematic of the experimental system used to change translation efficiency through mutations of the ATG context. (E) Measured expression distributions for two ATG context variants at three zinc induction levels shows that changing expression via induction or ATG context has a different effect on the shape of the expression distribution. Measured (F, squares) and fit (F, solid lines) of noise as a function of mean expression for three *ZRT1* promoter mutants (F, colors) that each has a unique four base-pair sequence immediately upstream of the ATG. A model (F, solid lines) in which the only difference between ATG context variants (different colors) is in the number of proteins produced per mRNA (B) fits the experimental data (squares) better than any alternative model (Figure S6).

To better understand what determines the relationship between expression and noise we used an analytical model of gene regulation (Figure 2A) [21] to predict changes in expression and noise in response to changes in TF activity (see Materials and Methods). We fit this model to measurements of *ZRT1* expression and noise and find that the model replicates our experimental results when an increase Zap1 activity causes an increase in the promoter on-switching rate (*Kon*) (Figure 2B,C). To further challenge the model we created a set of seven start codon context mutants of the *ZRT1* promoter (*NNNN*ATG) and measured the expression distribution of these variants at 12 different levels of TF activity (Figure 2D–F) (only three mutants are shown for clarity). These mutations change translational efficiency and therefore the number of proteins produced per mRNA (*b*), without affecting promoter dynamics (Figure S5). We find that ATG context variants at a single induction point differ in expression but not in noise, consistent with similar experiments in *B. subtilis* [14]. In support of the above hypothesis, we obtain the best fit of the model to our data when TF induction is modeled as changing *Kon* while ATG context variants change *b* (Figures S6 and S7). Furthermore, when fitting our model to data, we find that the optimal rate constants are on the order of experimentally measured promoter switching rates [9],[22],[23], and not TF binding/unbinding rates [24]. This suggests that promoter switching rates probably correlate with, and are partially determined by, TF concentration and binding kinetics. However, each TF binding event does not necessarily lead to transcription initiation. These results suggest that increases in TF activity increase the frequency of transcriptional bursts, while increases in translational efficiency cause an increase in the size (number of proteins produced) of each burst. We note that this is in contrast to observations in *E coli* [16] and in yeast at the *GAL1* promoter [25], in which TF induction appears to change the promoter off-switching rate (*Koff*) but consistent with measurements of the *PHO5* promoter [20].

### Repression of *ADH1* and *ADH3* by Zap1 Is Likely Due to a Decrease in the Frequency of Transcriptional Bursts

**Figure 3:**
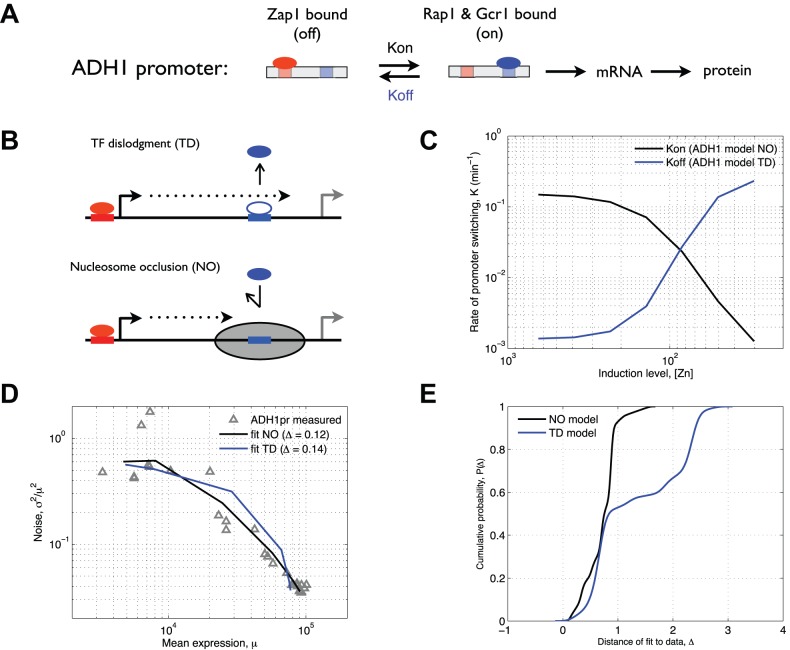
Measured and modeled gene expression for ADH1. We model *ADH1* expression using a two-state kinetic scheme (A) in which *Kon* and *Koff* are determined by the binding of transcriptional activators (blue circle) or a repressor (red circle). (B) Two mechanisms have been proposed for repression by upstream interfering transcription: TF dislodgment, in which an alternative transcript dislodges the bound activator, and nucleosome occlusion, where transcription through the promoter results in an occluding nucleosome that prevents binding of the activator. Hence, we assume that TF dislodgment increases the dissociation rate of the activator and that nucleosome occlusion results in a decrease in the binding rate of the activator. (C) We fit the model such that either *Kon* (black) for nucleosome occlusion or *Koff* (blue) for TF dislodgment changes as a function of [zinc]. (D) Measured mean expression versus noise (triangles) and fits (lines) of both model variants show that the nucleosome occlusion model has a better fit to the data (Δ is distance of fit to data). (E) To compare the robustness of each model, each parameter was independently perturbed 50 times over a 2-fold change from the fit value, and the distance of each model to the data was computed. Shown are the cumulative distributions of these distances. The narrower distribution of the nucleosome occlusion model (black) shows that it is significantly more robust to parameter variation than the TF dislodgment model (blue).

In addition to increasing expression of target genes, Zap1 can also act as a repressor. Zap1 represses two targets (*ADH1* and *ADH3*) by binding upstream of the core promoter and inducing intergenic transcription through the core promoter, probably promoting dissociation of the activating TF Rap1 (Figure 3A,B) [26]. Two mechanisms have been proposed for repression by transcriptional interference: dislodgement of TFs and the Pol II pre-initiation complex by RNA Polymerase [27], and competitive binding, one form of which is deposition of nucleosomes in the otherwise nucleosome-free region where the activating TFs and Pol II bind [28]. We hypothesized that deposition of nucleosomes would result in occlusion of the activating binding site, the TATA box, and Pol II binding, thus reducing the effective TF concentration and lowering the frequency of transcriptional activation. We model this mechanism as a reduction in *Kon*. Alternatively, passage of RNA polymerase may dislodge already bound Rap1, TBP, and/or the RNA polymerase pre-initiation Complex. This would shift the promoter from the “on” into the “off” state, thus reducing the length of each transcriptional on state and therefore the number of mRNA molecules produced during each transcriptional burst (Figure 3C). We model this mechanism as an increase in *Koff*. To determine the ability of dislodgement by Pol II (TD) or occlusion of TF binding by nucleosomes (NO) to explain our experiments, we fit each model to the data. We find that the NO model fits our data better than the TD model (Figure 3D) (see Materials and Methods). Furthermore, the NO model consistently fits the data better in the case in which we vary each parameter by up to 2-fold. The increased robustness (Figure 3E) and decreased sensitivity (Figure S8) of the NO model gives us further reason [29] to favor a model in which repression by Zap1 at the *ADH1* and *ADH3* promoters occurs by inducing intergenic transcription and nucleosome deposition over the core promoter and/or Rap1 binding site.

### *ZRT2* Achieves a State of Low Expression and Low Noise Due to a Repression-Mediated Mechanism of Intrinsic Noise Reduction

Uniquely among Zap1 target promoters, *ZRT2* responds nonmonotonically to an increase in Zap1 activity, whereby its expression first increases then decreases in response to increasing Zap1 activity [18]. In the activating regime of *ZRT2*, noise decreases as expression increases, suggesting a *Kon* (burst frequency) dominated change that is similar to the purely activated targets. However, in contrast to the repressed targets *ADH1* and *ADH3*, where noise increases with the decrease in expression, in the regime where *ZRT2* expression decreases noise remains constant. These results suggest that the decrease in *ZRT2* expression is a result of a decrease in burst size (see below), with the consequence of having induction points that have the same mean expression level but different expression distributions (Figure 1F). At high induction, the distribution is less noisy (Figure 1F, blue) than at low induction (Figure 1F, red). Thus, the ZRT2 promoter reaches a state that is unique amongst Zap1 targets that is characterized by both low expression and low noise. Taken together, these findings suggest that although *ADH1*, *ADH3*, and *ZRT2* are all repressed by Zap1, the mechanism by which *ZRT2* is repressed is unique.

### A Zap1 Binding Site Near the TATA Box Is Both Necessary and Sufficient for Repression Through Zap1-Mediated Burst Size Reduction

**Figure 4:**
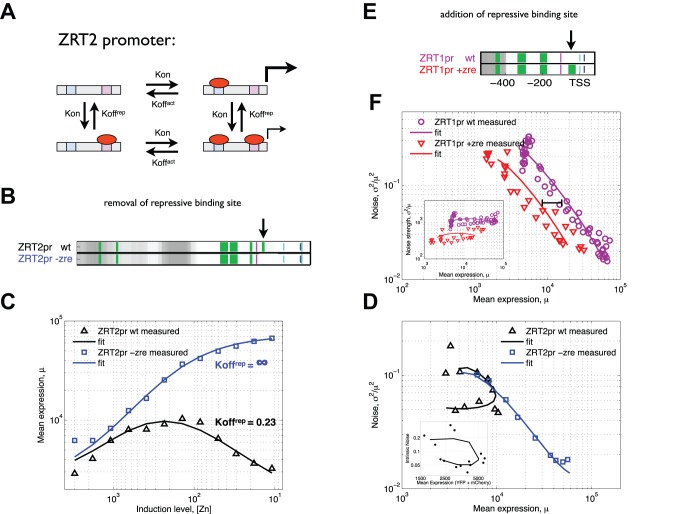
A repressive Zap1 binding site is both necessary and sufficient for repression in ZRT2. (A) We model *ZRT2* expression with a four-state kinetic scheme that represents four promoter configurations as a result of binding and unbinding of Zap1 to two different binding sites. One binding site is activating (blue square) and the other repressing (purple square), and as a result we assume that each configuration can have different transcriptional activity (see Materials and Methods for a detailed description of the model). (B) Promoter architectures are shown in terms of Zap1 binding sites (green), TATA box (purple), TSS (light blue), and nucleosome occupancy (white to grey for increasing occupancy) for wild-type *ZRT2* and a *ZRT2* mutant (−zre) in which the repressive Zap1 binding site was removed (at the arrow). (C) Measured (triangles and squares) and modeled (lines) mean expression as a function of [zinc] for wild-type *ZRT2* (black) and the −zre mutant (blue). (D) The same measured data and model from (C) are shown for mean expression versus noise. The *ZRT2* model was simultaneously fitted to the wild-type (C, D, black line) and the mutant (C, D, blue line) with the assumption that the only difference between wild-type and mutant is that the *Koff^rep^* of the mutant is infinite, to model the removal of the repressive binding site. Intrinsic noise (D, inset) measured in a dual reporter assay shows the same mean to noise scaling. Two biological replicates for each induction level are shown (points) with a smoothed line drawn through the induction points. (E) The promoter architectures are shown for the wild-type *ZRT1* promoter and a +zre mutant in which a repressive Zap1 binding sites was added around the TSS/TATA (at the arrow). (F) Measured mean expression and noise for the *ZRT1* wild-type (purple circles) and the +zre mutant (red triangles), and mean expression versus noise strength (inset). The *ZRT2* model was simultaneously fitted to both wild-type *ZRT1* (purple line) and +zre mutant (red line) again with the assumption that only *Koff^rep^* changes as a result of the addition of a repressive binding site. The black bar and inset indicate that a shift in expression occurred without a change in noise consistent with the assumption that the repressive binding site changes the apparent “off” rate and not the “on” rate.

In response to increasing Zap1, *ZRT2* expression first increases and then decreases. The activation by Zap1 is a result of Zap1 binding at activating binding sites 250–300 bp upstream of the start codon, while the repression is due to the presence of repressive Zap1 binding sites near the TATA box (between −90 and −112) [18]. We made a variant of the ZRT2 promoter (*ZRT2-zre*) that lacks the repressive binding sites (Figure 4B). We hypothesized that a model of the *ZRT2* promoter should include promoter states in which Zap1 is bound as an activator, as a repressor, and both as activator and repressor (Figure 4A). Based on experimental evidence [18], we model the binding site affinity for the repressive site as weaker than that of the activating site. We assume that binding of Zap1 to the repressive site turns off the promoter but does not affect the transition probabilities between states. When the model was simultaneously fit to both the *ZRT2-WT* and *ZRT2-zre* experimental data, we find that the model obtains a good fit to data when, like with *ZRT1*, an increase in Zap1 activity increases *Kon* and does not affect any other parameters. Interestingly, we find that the repressed state (state 4, Figure 4A) is not fully off, but has a small, but not insignificant, transcription rate relative to the transcription rate of the active state (state 2, Figure 4A). Notably the only parameter change required to change from ZRT2-WT to ZRT2-zre is setting *Koff^rep^* to be very high, mimicking the mutation of the repressive binding sites (Figure 4C,D). These experimental and modeling results suggest that binding of the transcriptional activator Zap1 to a binding site between the TATA box and TSS is necessary to generate a promoter state with low transcriptional activity. Notably, a very simple promoter model is able to replicate a nonmonotonic response to changes in TF activity. Furthermore, it suggests that *ZRT2* is able to reach a state of low expression and low noise purely through transcriptional regulation due to a promoter state with high burst frequency (due to binding of activating Zap1) and low burst size (due to binding of repressive Zap1).

These results suggest that in the *ZRT2* promoter, an increase in Zap1 both increases the frequency and decreases the size of transcriptional bursts. Therefore, our simple kinetic model shows that adding a repressive binding site for the activating TF is sufficient for explaining both *ZRT2* expression and noise as a function of induction.

Repression of *ZRT2* is accompanied by a decrease in noise strength, suggesting that repression occurs via a decrease in burst size. We therefore hypothesized that addition of a repressive Zap1 binding site to a native Zap1 target that lacks repression would cause a decrease in expression and burst size. To test this hypothesis, we added a consensus Zap1 binding site (ACCTTAAGGT) upstream of the transcription start site of *ZRT1* (Figure 4E, ZRT1pr+ZRE). Consistent with our hypothesis that this repressive site reduces expression through a decrease in burst size, this additional site results in a constant ∼2-fold decrease in expression, a decrease in noise strength, and no change in noise (Figure 4F). A model identical to the *ZRT2* model (Figure 4A), except that the repressive site has a higher affinity to Zap1 than the activating site, replicates the experimental data (Figure 4F). Interestingly, we find that while both models require the repressed state to be partially active, the repressed state of the *ZRT1* promoter has higher activity (in model and data) than for the *ZRT2* promoter. This may be because *ZRT2* has at least two repressive Zap1 binding sites, while we only introduced a single repressive binding site into *ZRT1*. Nevertheless, these results show that the presence of a Zap1 binding site between the TATA box and transcription start site is both necessary and sufficient for repression mediated by a decrease in burst size.

### Mutation of Additional Repressive Zap1 Binding Sites Suggests That a Combination of Activation and Repression May Be Common

**Figure 5:**
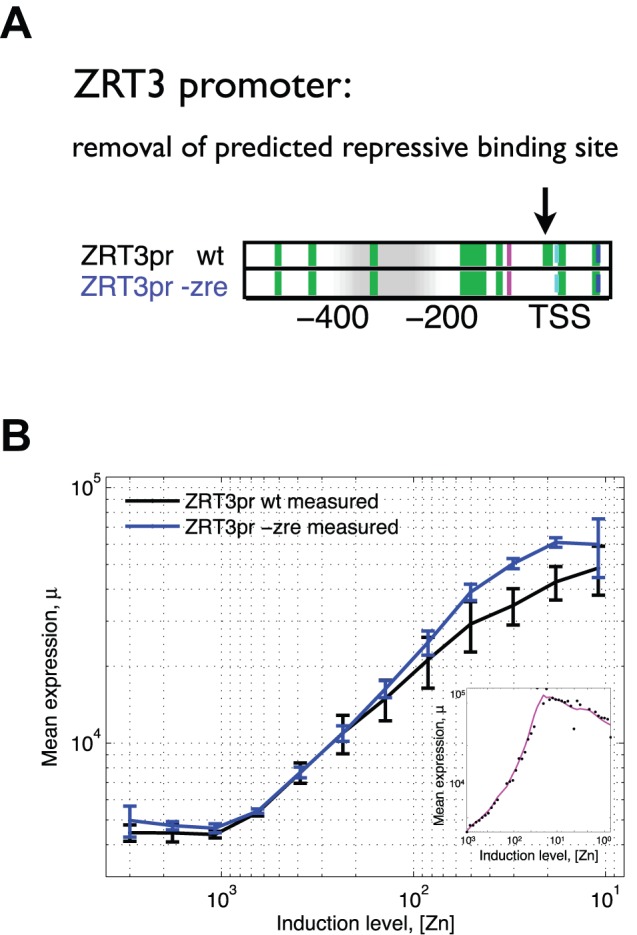
Removal of a predicted repressive Zap1 binding site increases expression of ZRT3. (A) Promoter architectures are shown for wild-type ZRT3 (wt) and a ZRT3 mutant (−zre) in which a potential repressive Zap1 binding site was removed (at the arrow). Shown are Zap1 binding sites (green), TATA box (purple), TSS (light blue), and nucleosome occupancy (white to grey for increasing occupancy). The potential repressive binding site was predicted using a bioinformatics search. (B) Consistent with this prediction mean expression is higher for the −zre mutant (blue) compared to the wild-type (black). The difference in expression appears only to exist at higher induction, consistent with the idea that repression is a function of Zap1 induction. Further induction of wild-type ZRT3, at very low zinc levels (inset), appears to decrease expression, consistent with a ZRT2-type repressive mechanism in which expression first goes up and then down with increasing TF levels.

A computational search for Zap1 binding sites between the TATA box and the transcription start site identified three weak Zap1 binding sites in the *ZRT3* promoter (Figure 5A). A closer look at the *ZRT3* induction curve at very low zinc concentrations showed that expression of *ZRT3* decreases slightly at high Zap1 induction (Figure 5B, inset). To determine if these weak Zap1 sites were functional, we mutated them and measured expression of the wild-type and mutant *ZRT3* promoters. Consistent with our hypothesis that Zap1 binding sites around the TSS are repressive, removal of the presumptive Zap1 binding sites increased expression (Figure 5B), in particular at higher induction, consistent with our model in which repression is a function of repressor activity. This suggests that low-affinity binding sites may be functional at high TF concentration, perhaps mostly at promoters that have additional high-affinity binding sites.

### Activation and Repression by the Same TF as a Mechanism for Reduction of Extrinsic Noise Due to Fluctuating TF Levels

**Figure 6:**
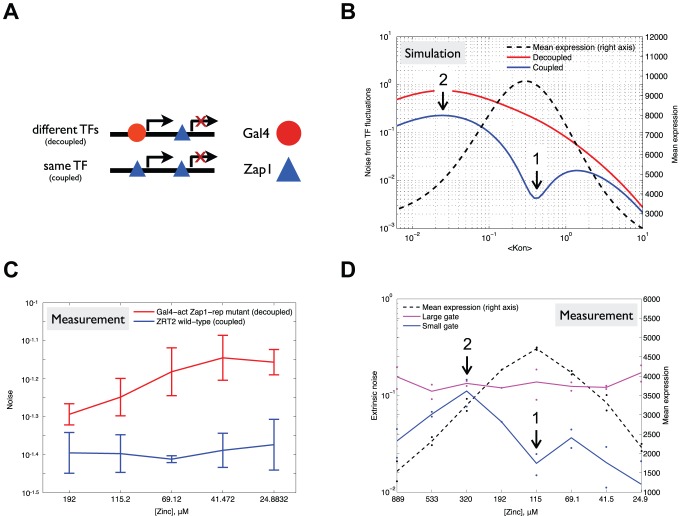
Activation and repression by the same TF as a mechanism for noise reduction. (A) A promoter that is both activated and repressed can be regulated by two different TFs (decoupled; e.g., Gal4-act and Zap1-rep) or one TF (coupled; e.g., Zap1) that functions as both an activator and repressor. (B) A simulation of noise as a result of fluctuations in TF concentration is shown for a coupled (blue) and decoupled (red) system. The *y*-axis shows noise as a result of TF fluctuations as a function of promoter induction (mean on-switching rate, Kon) for the coupled (blue) and decoupled (red) system. In addition, the mean expression at each induction level is shown (dashed line). Noise from TF fluctuations was quantified by sampling the model at different TF concentrations (i.e., Kon values) that were drawn from a gamma distribution (see Materials and Methods for a detailed description of the model). The model predicts that coupling of activator and repressor (e.g., if they are the same molecule) reduces noise. Notably, reduction is maximal where mean expression peaks (arrow 1). (C) Noise measurements, at various zinc induction levels, of native ZRT2 (blue) and a mutant that has two Gal4 UASs upstream of a repressive Zap1 site (red). The coupled system (wild-type Zrt2) has consistently lower noise than the decoupled system (Gal4-act Zap1-repr), as is predicted by our model. (D) Measurement of extrinsic noise from a dual-reporter assay is shown as a function of zinc induction. Nonstringent gating on cell size (through forward and side scatter) shows an extrinsic noise that is constant with induction (purple). However, strict gating (through a small forward and side scatter gate) significantly reduces the extrinsic noise and reveals a signal that changes with zinc induction (blue). We hypothesize that this signal is determined by noise from TF fluctuations, which according to our model has specific behavior as a function of induction. As predicted by our model we find a reduced noise where mean expression (dashed line) is maximal and sensitivity to TF changes is minimal (B, D, arrow 1), and minimal reduction (maximal extrinsic noise) where mean expression is most sensitive to changes in TF concentration (B, D, arrow 2).

The ZRT2 promoter presents a case in which the activator and repressor are the same TF. We were intrigued by this mechanism and wondered whether this affects the noise properties. Many promoters in yeast are regulated by the binding of both activators and repressors to different binding sites in the promoter [30]. The activator and repressor can be different proteins (e.g., *ADH1* is activated by Gcr1 and Rap1 and repressed by Zap1) or the same protein (such as ZRT2 that is both activated and repressed by Zap1) (Figure 6A). We hypothesized that the sensitivity to TF fluctuations for a promoter that is both activated and repressed depends on the coupling between activator and repressor. For example, we expect that when activation and repression are done by the same TF, in a regime where a change in activator binding has the exact opposite result on expression as the same change in repressor binding, the promoter is insensitive to any fluctuations in TF levels. This is because any random fluctuation in the concentration or activity of the TF will have an equal activating and repressive effect and therefore result in no net change in target activity. To study this hypothesized phenomenon, we used our kinetic model of *ZRT2* and simulated the case where activator and repressor are different (decoupled) and where they are the same TF (coupled). We then calculated the contribution of TF fluctuations to expression noise throughout the induction (Figure 6B) (see Materials and Methods for a detailed description of the model). Coupling of the activator and repressor reduces the sensitivity to TF fluctuations throughout induction and places the point of minimal sensitivity to TF fluctuations at the point of maximum target gene expression (Figure 6B, blue line).

Our model predicts that the total sensitivity to TF fluctuations is reduced throughout the induction curve, and that this reduction is greatest at maximal promoter expression (Figure 6B, point 1). To test this we measured extrinsic noise (the contribution of variance in all factors; e.g., ribosomes, Zap1, Pol II) for the native ZRT2 promoter using a dual-reporter. We find that extrinsic noise is constant across the induction (Figure 6D, purple, see also Text S1 and Figure S11). However, when we remove as much global extrinsic noise [4] as possible using a very narrow forward and side scatter gate (Figure S9) [25] we hypothesize that we are left with mostly pathway-specific noise (e.g., noise due to TF level fluctuations). In support of this hypothesis, we find that pathway-specific noise is not constant, but rather varies greatly (around 10 fold) with induction. We find that this signal, which we expect to be dominated by changes in TF sensitivity, does indeed drop around the point of maximal expression (Figure 6D, blue), consistent with our model. In fact, the extrinsic noise replicates quite well the general predicted change in TF sensitivity with induction.

**Figure 7:**
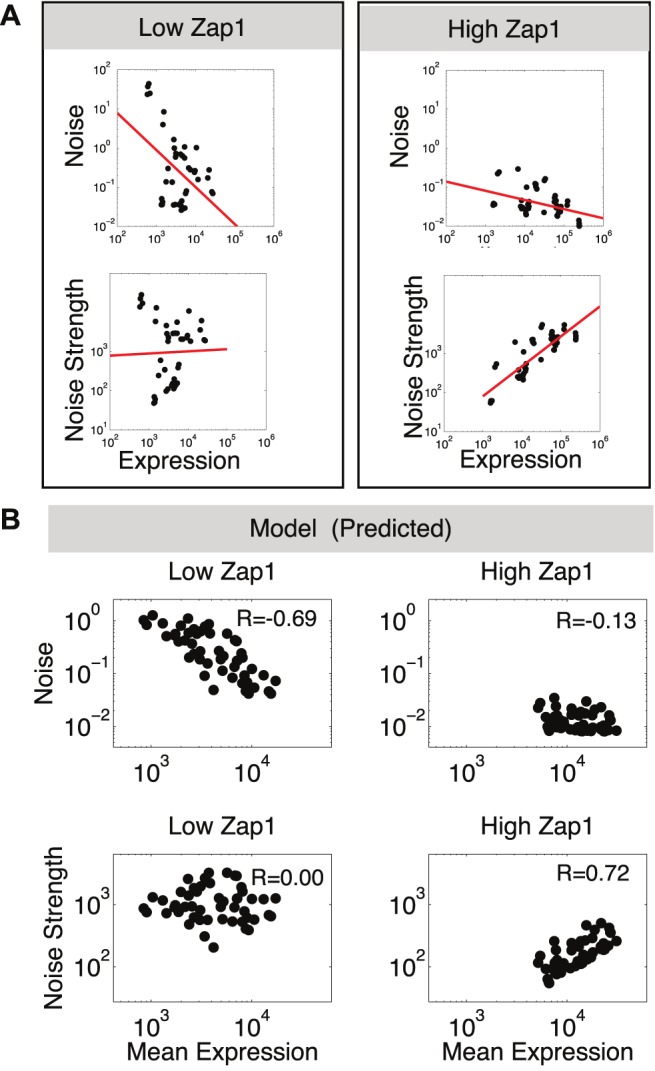
The correlation of noise and noise strength with expression changes with TF concentration. (A) Scatter plots of noise (top) and noise strength (bottom) graphed against expression for each promoter at low (left side) and high (right side) Zap1 induction points. A line fit to each set of points using linear regression shows that, across promoters, noise strength is uncorrelated with expression at low TF concentration, but is positively correlated with expression at high TF concentration. (B) Noise and noise strength graphed against expression for high and low TF as in (A) but for *in silico* promoters that differ in both *K_ON_ and K_TL_*. The change from low to high TF was simulated by multiplying the initial *K_ON_* of each promoter by 20.

Finally, our model predicts that decoupling of activator and repressor will increase total noise as the sensitivity to TF fluctuations is increased. To test this we replaced the two activating Zap1 binding sites of *ZRT2* with two Gal4 binding sites (Figure 6A) and measured expression and noise throughout the repressive regime at high Gal4 induction (0.5% galactose) (Figure S10). Consistent with our model, the Gal4-Zap1 regulated *ZRT2* variant has higher noise than the wild-type promoter (Figure 7C). These results show that, while repression is able to reduce expression and keep noise constant (Figure 4), a transcriptional regulatory motif, in which the activator and repressor are the same protein, is capable of reducing noise even further. This suggests that the coupling of activator and repressor can be a mechanism to regulate gene expression with less variability.

### The Dominant Source of Differences Between Promoters in Expression and Noise Changes with TF Concentration

We hypothesized that the source of differences in expression between genes might change with TF concentration. At low TF concentrations, promoters will be inactive most of the time, and differences in expression may depend mostly on differential recruitment of the TF. In this case, the major source of differences in expression between promoters should stem from the frequency with which transcriptional bursts occur. Alternatively, at saturating concentrations of activating TF, the promoter should be “on” most of the time and the major difference in expression between promoters should arise from the transcription and translation rates of each promoter. Thus, as the concentration of TF changes from negligible to saturating, we expect the transcription and translation rates of each promoter to become more important in determining expression differences between genes.

To determine whether burst frequency or burst size dominate the differences in expression between promoters, for each induction level, we measured the correlation between expression and noise or noise strength across promoters. Consistent with the above hypothesis, across all promoters, noise is highly correlated with expression at low levels of Zap1 activity (*R* = −0.66, *p*<0.01), while noise strength is uncorrelated (*R* = −0.02, *p*<0.94) (Figure 7A). This suggests that at low TF concentration, burst frequency determines the differences in expression across promoters. Conversely, at high levels of Zap1 activity, noise strength is correlated with expression (*R* = 0.63, *p* = 0.01), and noise is slightly less correlated (*R* = −0.55, *p* = 0.04) (Figure 7A). Overall, we found a continual increase in the correlation between noise strength and expression with increasing TF activity (unpublished data). To test the hypothesis that these differences are due to a change in the dominant source of expression difference between promoters, we generated 50 random genes *in-silico* that differ only in their rates of promoter on-switching (*K_ON_*) and translation (*K_TL_*). We then performed an induction by increasing *K_ON_* for each promoter to 20 times its original value. This results in a mean to noise and mean to noise strength scaling that is strikingly similar to what we observed for the native Zap1 targets (Figure 7B). Taken together, our results suggest that as a set of targets of the same TF are induced, the major source of expression differences between them changes from being dominated by burst frequency to a combination of burst frequency and burst size.

## Discussion

We have measured the dose response curve, in terms of expression and noise, for a set of native yeast promoters that are all targets of the same TF, yet are regulated by that TF via at least three distinct transcriptional mechanisms: activation, repression by binding between the TATA box and TSS, and repression by induction of an upstream interfering transcript. Although noise generally decreases with increased expression, the quantitative scaling of noise with expression is specific to each promoter and depends on the mechanism by which the TF regulates the promoter.

### The Promoter Sequence Determines How Activation by Zap1 Affects Noise and Expression

Similar to the global trend [3], our data suggest that changes in expression of individual promoters are dominated by differences in burst frequency. This is consistent with Zap1 binding to promoters being limiting for transcriptional activation, especially at low Zap1 concentrations, and with the proposal that the rate-limiting step in transcription for yeast is promoter firing rate, which is determined by TF search times [31]. However, the observation that different activated targets have different scaling between noise and expression suggests that while activation by Zap1 acts only through burst frequency at most activated promoters, it may act partially or even completely through burst size at other activated promoters. This is entirely reasonable; Zap1 is not the only TF acting at these promoters, and the promoters differ in both nucleosome organization and the presence and location of TATA boxes. Experiments that placed a tetO sequence at different locations within the *FLO11* promoter suggest that the same TF can have different effects on promoter dynamics, depending on the location of binding sites within the promoter [23]. Unfortunately, there are not enough strongly induced Zap1 targets in *S. cerevisiae* to identify the promoter architecture features that determine the source of the promoter-specific slope. It will be interesting to perform dose-response curves for a larger set of promoters from other yeasts, or on synthetic promoters, in order to identify promoter architectures that determine the promoter-specific slope.

### Different Mechanisms of Regulation by the Same TF Can Cause Similar Changes in Expression But Different Changes in Noise

Our observation that repression by production of an upstream interfering transcript causes an increase in noise, while repression when the TF binds near the TATA box causes a decrease in noise, suggests that different dynamics occur at each promoter during repression. This, along with previous observations [18],[23],[25],[28],[32], suggests that the mechanism of regulation by any TF is determined in cis by the promoter architecture. Binding sites between the TATA box and TSS decrease burst size, binding sites within a few hundred bases upstream of the TATA box increase burst frequency, and binding sites further upstream, with a nearby downstream TATA box, repress through a reduction in burst frequency. These data show, to our knowledge, for the first time that different promoter architectures can cause a similar change in expression in response to changes in TF activity, but exhibit different changes in noise.

### High Burst Frequency and Low Burst Size Is a Strategy to Produce Low-Abundance Proteins with Low Noise

If the genome-wide scaling of expression and noise extends to proteins with very low expression, then a large fraction of cells will have zero molecules of protein [33]. Single-molecule studies have confirmed this: many cells have zero molecules of proteins with low levels of expression [4]. However, many proteins expressed at low levels are essential. This raises the question: How does the cell maintain a low level of both expression and noise for essential proteins, so that all cells have the minimum number of proteins? Our results showing that burst size regulation can reduce expression without increasing noise suggest a way out of this trap. Lowly expressed genes tend to be bound by many transcriptional regulators, both activators and repressors [30]. Low levels of an activating TF result in low expression and high noise. Notably, a motif in which weak transcription but efficient translation generates high noise may exist at the *comK* gene in *B. subtilis* [34]. In contrast, combinatorial regulation that results in high burst frequency and low burst size (approaching the Poisson limit [9]) provides a regulatory motif through which cells can produce low levels of protein with low cell-to-cell variability. Our identification of this same regulatory motif in the *ZRT3* promoter suggests that this motif may be common. This regulatory strategy may be used to prevent some cells from having zero molecules of protein when expression is low.

### Coupling of Activator and Repressor as a Mechanism for Reducing Extrinsic Noise

The concentrations of TFs, like those of all other proteins, vary greatly from cell to cell. We expect that these variations have a significant impact on the cell-to-cell variability of target gene expression [4], and therefore wondered how cells deal with this source of noise. Interestingly, we find that ZRT2 is able to reduce noise through its reduced sensitivity to fluctuations in TF levels, as a result of activator and repressor being the same molecule. Mechanisms for extrinsic noise reduction have been previously reported [21]. However, to the best of our knowledge, we are the first to propose theoretically and confirm experimentally a mechanism for desensitizing promoters to TF noise. Noise as a result of TF fluctuations has been proposed theoretically in several studies [4],[35]. In fact, Bai et al. propose a dual-reporter experiment to investigate extrinsic noise resulting from TF fluctuations, which we have performed in this work (Figure 6D). We note that noise from TF fluctuations is a special case of noise propagation in a gene network, where the noise of a downstream gene is a function of its intrinsic noise and the noise from any upstream genes [36]. An alternative mechanism for a similar reduction in sensitivity would be the regulation by multiple different decoupled TFs. We hypothesize that as the number of different TFs increases, target sensitivity (and therefore noise) decreases, if the TFs are sufficiently de-correlated. This potential mechanism, as well as the general characterization of the effect of TF noise on target noise, would make the subject of a meaningful follow-up study.

### The Dominant Type of Noise Changes with TF Concentration

The observed change in the scaling between noise and expression throughout the increase in TF concentration suggests that variability between promoters in burst size (transcription efficiency, translation efficiency, and promoter off-switching rate) becomes more important as TF concentration is increased. This suggests that differences in promoter architecture play different roles at low and high TF concentrations. In the presence of limiting TF, promoter architecture may determine expression by determining TF search time, through the number of accessible TF binding sites. However, at high TF concentration, promoters are mostly bound by TFs, and the transcription and translation efficiency of each gene may play a greater role in determining expression. This idea is supported by the positive correlation between noise strength and expression at high TF concentration, as would be expected from theory [20]. In addition, differences in burst frequency cannot account for the measured single-cell expression distributions at high TF concentration. These data suggest that the dominant sources of gene-to-gene variability in expression change with TF concentration: at low TF concentration burst frequency (the ability of the promoter to recruit TF) differences dominate, whereas at high TF concentration burst size (transcriptional and translational efficiency) differences dominate.

Overall, our results show that the relationship between expression and noise is highly dependent on the promoter architecture. One implication of this finding is that using only a single TF, evolution can implement diverse expression profiles with unique noise properties. The fact that repression of *ZRT2* by Zap1 is evolutionarily conserved suggests that there is an advantage to this ability.

## Materials and Methods

### Yeast Strains

Construction of promoter-YFP strains was performed as described previously [19]. In brief, a master strain, *his3*::TEF2pr-mCherry-YFP-NatMX4, was created in the background strain Y8205 [37] by homologous recombination. Each promoter-YFP strain was created by integration of a PCR product containing the native promoter along with URA3 as a selection marker. Integration by homologous recombination upstream of YFP was confirmed by DNA sequencing and by identical expression and growth of multiple independent transformants.

### Creation of Promoter Variants

To introduce, alter, and remove elements within ∼150 bp of the ATG, we developed a method in which an existing URA3-promoter-YFP cassette is amplified over multiple rounds of PCR. In each subsequent round, a new primer is used that further extends the product towards the YFP and optionally introduces designed mutations. Thus multiple site-directed mutations can be tiled onto the 3′ end of the promoter. All promoter variants were confirmed by DNA sequencing.

### Yeast Growth and Expression and Noise Measurements

Yeast strains were grown overnight to saturation in YPD, resuspended in low zinc medium [18], and grown overnight to saturation in media lacking zinc. Cultures were then diluted 1∶40 in water and 6 µl of this dilution was inoculated in 130 µl of low zinc media supplemented with various concentrations of zinc. Cells were grown in round-bottom 96-well plates shaking at 30°C a minimum of 12 h, to approximately 5*10^6^ cells/ml prior to expression measurements. For galactose inductions, cells were pregrown overnight to saturation in low zinc media with 0.5% galactose as the sole carbon source to induce expression, then resuspended in media with varying concentrations of zinc with 0.5% galactose similar to the above experiments. Flow cytometry was performed on a BD LSRII. YFP and mCherry were excited using 488 nm and 561 nm lasers and emitted light was collected with 525/50 nm and 610/20 nm band-pass filters, respectively. There is no detectable spillover of YPF or mCherry into the other channel using these filters and lasers. Expression and noise measurements were collected and calculated using the ratio of YFP over mCherry for each cell. To obtain expression and noise measurements from each well, a relatively homogenous subpopulation of mostly G1 cells was chosen by gating on forward and side scattering. Wells containing fewer than 500 cells after gating or with obvious contamination were excluded from further analysis. Noise was quantified as the variance over the mean squared and noise strength as the variance over the mean.

### Kinetic Model of Activation and Repression

We model stochastic promoter state switching, transcription, and translation using the master equation following the approach described by Sanchez et al. [38], which in turn is an adaptation of previous derivations of the master equation for gene regulation [38]–[40].

In this description of promoter regulation, TF binding and unbinding events determine the transitions between promoter states. A change in transcriptional activity occurs when a transition is made to a state with differing transcription rate. Each promoter state is modeled to have a low (including zero) or relatively high transcription rate to describe in-active (“off”) or active (“on”) states, respectively. Translation occurs in bursts with the probability of a burst described by a geometric distribution. The master equation (in matrix notation) takes the form:
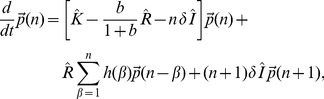
(1)where 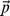 is the vector of probabilities of having *n* proteins in the cell for each promoter state. 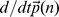 describes the time evolution of these probabilities. 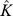 is the matrix of promoter state transition rates, where 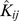 is the rate of transitioning from state *j* to state *i* and 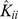 is −1 times the sum over all outgoing rates from *i*. 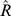 is the diagonal matrix of transcription rates with 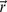 on the diagonal (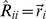), where 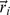 is the transcription rate of state *i*. 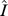 is the identity matrix. *b* is the average burst size (proteins produced per mRNA). δ is the protein degradation rate. *h*(β) describes a geometric distribution and is the probability of producing a burst of size *β*.

To derive the mean protein abundance and variance, we solve this system at steady state, thus for 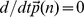, we get mean protein abundance:
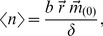
(2)where 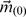 is the zeroth partial moment of the distribution of mRNA abundance and is the solution to:
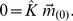
(3)


We can get noise (σ^2^/μ^2^) and noise strength (σ^2^/μ) by deriving:
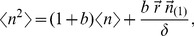
(4)where 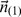 is the first partial moment of the distribution of protein abundance and is the solution to:
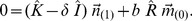
(5)


Variance (σ^2^) is:
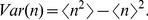
(6)


Therefore, noise (σ^2^/μ^2^) becomes:
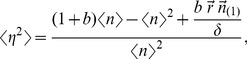
(7)and noise strength (σ^2^/μ):
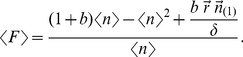
(8)


We solve the master equation for a number of different promoter architectures, where we define 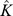 and 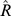 for each system to describe the specific promoter states, the transitions between them, and the transcriptional activity of each state.

#### Case ZRT1

We describe gene expression and regulation of ZRT1 using a two-state kinetic scheme that represents switching between an active (ON) and inactive (OFF) promoter configuration. We assume that on switching (with rate *Kon*) and off switching (with rate *Koff*) are a function of binding and unbinding, respectively, of the Zap1 transcriptional activator at the ZRT1 promoter. The Zap1 bound (ON) state is transcriptionally active (with rate *r1*) and we allow the unbound (OFF) state to have some (leaky) transcriptional activity (with rate *r2*). 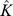 and 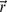 thus become:
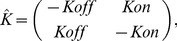
(9)

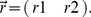
(10)


The promoter off rate (*Koff*) is a function of Zap1 binding affinity and therefore assumed to be constant. We describe the promoter on rate (*Kon*) with a Hill-equation (see Eq. 11) with *K_min_* and *K_max_* as the minimum and maximum possible rates, respectively, *[Zn]* as the zinc concentration (induction level), *[Zn]*_mid_ as the *zinc* concentration that gives half maximal induction, and *H* as the Hill-coefficient (i.e., the sensitivity of *Kon* to changing zinc).
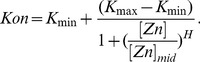
(11)


#### Case ZRT2

ZRT2 is both activated and repressed by Zap1. We model Zap1 binding (*Kon*), unbinding at the activating binding site (*Koff^act^*), and unbinding at the repressive binding site (*Koff^rep^*). We describe *Kon* with a Hill-equation as a function of [*Zinc*] (see Eq. 11). Transcription can occur from each state (i.e., expression is leaky), however we assume that the state where the activator, and not the repressor, is bound has the highest transcriptional activity (with rate *r1*). 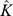 and 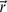 therefore become:
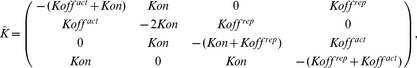
(12)

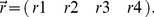
(13)


#### Case ADH1

ADH1 is activated by Rap1 and repressed by Zap1. We model the switching between the active (Rap1 bound) and repressed (Zap1 bound) states with the two-state kinetic scheme that we used to model ZRT1 activation by Zap1. Repression of ADH1 by Zap1 occurs through (one of) two hypothesized mechanisms: nucleosome occlusion and TF dislodgement. We model both mechanisms by a subtle difference in the dynamics of repression.

#### Case ADH1, Nucleosome Occlusion

Zap1-mediated intergenic transcription may repress ADH1 by causing nucleosome deposition in the otherwise nucleosome-free core promoter region, thus preventing the activator (Rap1) or PolII from binding. We model this mechanism by changing *Kon* as a function of induction, as occlusion (accessibility) effectively changes the on-switching rate of the promoter. More specifically we model *Kon* as a sigmoid that is a function of [Zinc] (see Eq. 14), where increasing zinc increases *Kon* (as Zap1 decreases) while *Koff* is constant.
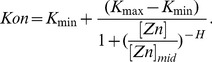
(14)


#### Case ADH1, TF Dislodgement

In the hypothesized TF dislodgement mechanism, repression occurs as the interfering transcript (caused by upstream Zap1 binding) dislodges the already bound activator (Rap1) or the PolII holoenzyme. This would effectively change the rate at which the promoter switches from on into the off state; hence, we model this by changing *Koff* as a (sigmoidal) function of [Zinc] (see Eq. 15), while keeping *Kon* constant.
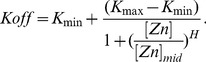
(15)


### Model Fitting and Robustness Analysis

We fit the kinetic scheme's analytical solutions of mean and noise of protein abundance to the measured mean and noise of fluorescence intensity (see Text S1 for a detailed description of the fitting procedure and parameter constraints). The goodness of fit is measured by the root mean squared error (distance, Δ) of both mean and noise.

To investigate the hypothesized effect of promoter mutations, we simultaneously fit the model to wild-type and mutant promoters while only one parameter is allowed to change between the fits.

We distinguish between two hypothesized ADH1 regulatory mechanisms by fitting two models to the measured data. While ADH1 nucleosome occlusion gives a better fit than TF dislodgement, both models have a good fit to the data. To investigate if nucleosome occlusion gives a significantly better fit to the data, for each fit found by optimization, we perform 2-fold perturbations on each parameter. By looking at the distribution of fits after perturbation, we get an idea of which model is more robust and as a result is more likely to be the correct model [29],[41]. We find that the NO model is significantly more robust than the TD model.

### Sensitivity Analysis of the Kinetic Model

To measure the sensitivity of the kinetic model to variations in each parameter, we performed a rigorous sensitivity analysis procedure described by Marino et al. [42] that uses the Latin Hypercube Sampling–based Partial Rank Correlation Coefficient (LHS-PRCC). First, we uniformly sampled 10,000 instances of the model (without fitting), each with a unique parameter setting, sampled from the entire allowed parameter space using Latin Hypercube sampling, and evaluated each of these models by measuring the distanced to the experimental data. Next, we calculate the Partial Rank Correlation Coefficient of the parameter value to the model score (goodness of fit) to measure the sensitivity of that parameter. We find that the NO model is significantly less sensitive to parameter variation than the TD model (see Figures S7 and S8 for sensitivity analyses of the ZRT1 and ADH1/3 models, respectively).

### Simulating Coupled and Decoupled Activation and Repression

To investigate the effect on gene expression noise of activation and repression by the same TF (coupled) versus activation and repression by two different TFs (decoupled), we extended the *ZRT2* kinetic model to incorporate fluctuations in the concentration of TF. We use a kinetic scheme in which on-switching rates for activator and repressor can be changed independently (*Kon^act^* and *Kon^rep^*, see Eq. 16). These rates are determined by the distributions of activator TF and repressor TF, respectively. We therefore assume that the on-switching rates have Gamma distributions with a constant shape parameter (burst size) and varying scale parameter (burst frequency) as the activator and repressor are induced. The means of the on-switching rates were chosen to be in the range of our model fits (10^−3^ to 10^1^), which are in accordance with previously determined promoter switching rates [9],[22],[31]. Next, we calculate the shape parameter of the distribution of *Kon* using the ratio between the mean of the measured protein distribution and the chosen mean of the on-switching rates (ratio of ∼10^4^), which we apply to the measured noise strength (∼10^3^). This gives a shape parameter value of around 10^−1^. Because the product of shape and scale is equal to the mean, we can compute the values of the scale parameter (10^−2^ to 10^2^). We note that the qualitative result of predicted noise reduction (Figure 7B) is robust to 10-fold changes (up and down) of both shape and scale parameter of the distributions of *Kon^act^ and Kon^rep^*. Finally, to simulate coupled and decoupled activation and repression, we sampled the on-switching rates of the activator and repressor from a bivariate gamma distribution with a normalized covariance of zero (decoupled) or one (coupled). Each sample represents a single cell with some amount of activator and repressor, and therefore some *Kon^act^* and *Kon^rep^*. We then computed the mean expression for each “cell” using the analytical solution of the ZRT2 model and calculated the predicted noise that results from fluctuations of activator and repressor as the squared coefficient of variation (sensitivity to TF fluctuations, η^2^_TF_).
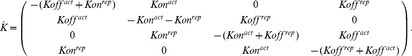
(16)


## Supporting Information

Figure S1 Zap1-regulated targets change expression in response to changes in zinc concentration. Shown is measured mean expression for four different Zap1 targets (*ZAP1*, *ZRT1*, *ADH4*, and *TKL2*) showing quantitatively different basal (high zinc, low Zap1) expression levels as well as different induction curves. Also shown is expression of YFP from a truncated *ENO2* promoter that lacks the two Zap1 binding sites, and is thus insensitive to changes in zinc concentration. For all YFP data, error bars show the standard deviation from at least three biological replicates. Shown in red is measured expression of TEF2pr-mCherry from all of the data from all strains shown.
(EPS)

Figure S2 Predicted Zap1 occupancy of each promoter is predictive of each activated promoter's change in expression in response to increasing Zap1. The range of expression (lowest to highest measured values) graphed against the predicted number Zap1 molecules bound to a 500 bp window from −600 to −100 relative to the transcription start site for each promoter. A thermodynamic model of promoter TF occupancy [37] shows that the measured Zap1 dose-response curve for each promoter highly correlates with the predicted occupancy of Zap1 at each promoter, suggesting that the number and affinity of Zap1 binding sites plays a large role in determining each promoter-specific dose-response curve. To identify sequence features in each promoter, we used a thermodynamic model in which Zap1 binding along the promoter sequence is determined by the concentration of the TF, its measured sequence specificities, and competition with nucleosomes [37].
(EPS)

Figure S3 The correlation of noise and noise strength with mean expression for all measured Zap1 targets throughout the induction. The correlation between expression and noise (A) and expression and noise strength (B) is shown for all measured expression data for each Zap1 target promoter. For each target, expression is normalized between zero and one. The mean Spearman correlation coefficient for all promoters is shown.
(EPS)

Figure S4 Gene-specific slopes are significantly different from each other. Distributions show the mean-noise slopes obtained by bootstrapping all biological replicate experimental measurements of noise (η^2^) and mean expression (μ) and fitting the line η^2^ = cμ^k^ 1,000 times for each promoter. The distributions show that the slope (*k*) values are significantly different and that slope value estimation is robust for most promoters.
(EPS)

Figure S5 Only part of the change in expression due to ATG context variants can be explained by changes in mRNA level. Shown are the fold differences in protein and mRNA between start codon context variants of the ZRT1 promoter. In order to determine if changes of the four nucleotides upstream of the ATG lead to differences in mRNA levels, we performed RT-qPCR on three ATG context variants plus the WT ZRT1 promoter. Because mCherry is expected to be constant between the different strains, YFP/mCherry ratios are used as measurements for both fluorescence and mRNA. In addition, because both measurements are in arbitrary units, we cannot compare numeric values directly. However, both measurements are linear, and therefore ratios relative to a common control (the CTTT strain) can be compared. We find a 2.1-fold change in protein levels and a 1.3-fold change in mRNA levels. In addition we observe no correlation between mRNA and protein. These results are consistent with previous data [43] showing that the start codon context can change protein expression without affecting mRNA levels.
(EPS)

Figure S6 A model in which zinc concentration changes promoter *Kon*, and ATG context variants change *b*, best explains the experimental data. There are many possible regulatory mechanisms by which zinc concentration and ATG context may change expression of ZRT1. In order to determine which regulatory mechanism best fits our data, we fit the model represented by the kinetic scheme in Figure 2A to our data 12 times. In each time, we mandated that a different pair of regulatory mechanisms be used to fit the induction of the ATG context variants. We find that a model in which the induction increases *Kon* and ATG context variants change *b* (first column) obtains the best fit to data. We note that similar regulatory mechanisms in which ATG context variants change burst size (columns 2 and 3) obtain fits that are almost as good. In contrast, a model in which zinc changes promoter off switching rate (*Koff*) never obtains as good a fit to the data, and is only capable of obtaining a reasonable fit to the data when ATG context variation changes the rate of transcription.
(EPS)

Figure S7 Sensitivity analysis of the ZRT1 model. To determine how sensitive the ZRT1 model is, we used LHS-PRCC (see Materials and Methods). We find that the fit of the model to data is highly sensitive only to *S*, a scaling factor we use to convert measured YFP/mCherry value per cell to number of YFP protein molecules per cell. (A) Density scatter plots from LHS sampling of the parameter space show how the fit to data (*y*-axis) changes as a function of each parameter (*x*-axis). Correlations of the data in (A) are shown together for comparison in (B).
(EPS)

Figure S8 Sensitivity analysis of both proposed ADH1 models shows that the nucleosome occlusion (NO) model is less sensitive to parameter variation than the TF dislodgment (TD) model. To determine how sensitive each of the ADH1 models are, we performed LHS-PRCC sensitivity analysis. (A) Density scatter plots from LHS sampling of the parameter space show how the fit to data (*y*-axis) changes as a function of each parameter (*x*-axis). Correlations of the data in (A) are shown together for comparison in (B). The NO model is far less sensitive to variation in biological parameters.
(TIF)

Figure S9 Gating of the ZRT2pr dual reporter causes a 10-fold reduction in extrinsic noise. For each cell, mCherry is plotted against YFP for one induction point from the *ZRT2pr* dual reporter. Extrinsic noise was reduced by gating using either a very wide (black) or very narrow (red) gate on forward and side scatter.
(EPS)

Figure S10 Mean expression and noise of decoupled activation and repression. The decoupled Gal4-activating Zap1-repressive mutant was measured at 0.5% galactose and varying Zinc concentrations. Shown is mean expression (black dashed line) and noise (magenta and blue lines), as a function of [Zinc]. Removing extrinsic noise (from magenta to blue line) to reveal the pathway specific and intrinsic noise by stringent gating decreases noise but does not show a noise reduction at specific induction levels.
(EPS)

Figure S11 Equivalence of YFP and mCherry distributions in the repressive regime of *ZRT2pr* expression. (A) In order to test the equivalence of the YFP and mCherry fluorescent reporters for use in a dual-reporter system, we compared the fluorescence distributions at different induction points. (B) A Kolmogorov-Smirnov test in which we sampled 500 cells from each induction point by bootstrapping shows that at low induction points the distributions are not equivalent, and therefore the measurement of intrinsic noise may not be valid. However, at induction points less than 600 µM, the distributions are equivalent.
(EPS)

Table S1 Lengths of cloned promoters inserts. Listed for each promoter is the length of each cloned sequence, in bases upstream of the ATG.
(XLS)

Text S1 This file contains supporting information.
(DOCX)

## Abbreviations

TF: transcription factor
TSS: transcription start site
