# Supplementary figures and images for: Promoter Sequence Determines the Relationship between Expression Level and Noise
Source: PLoS Biol. 2013 Apr 2;11(4):e1001528. doi: 10.1371/journal.pbio.1001528 (PMC3614515; doi:10.1371/journal.pbio.1001528)

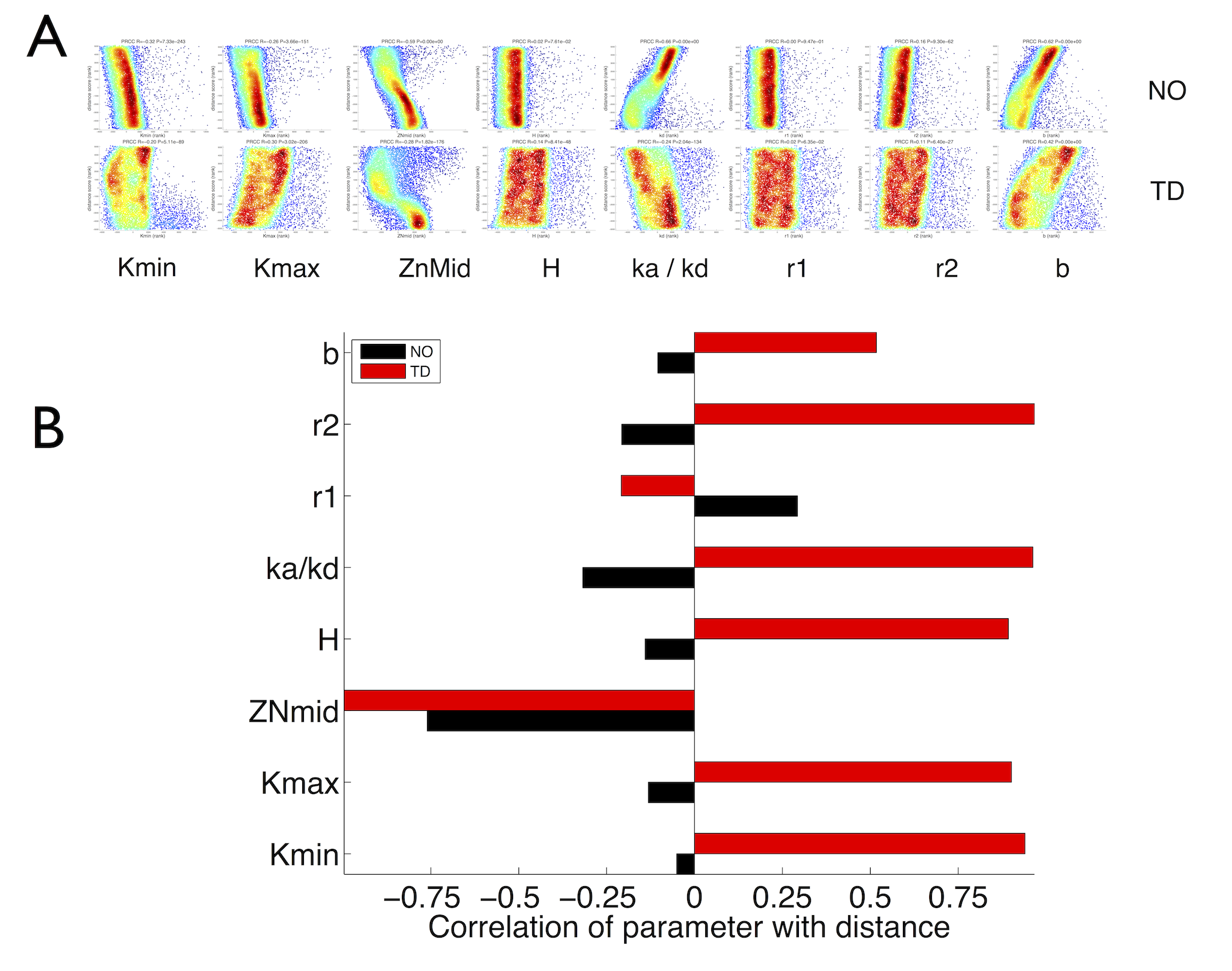

Supplement: Figure S8 — Sensitivity analysis of both proposed ADH1 models shows that the nucleosome occlusion (NO) model is less sensitive to parameter variation than the TF dislodgment (TD) model. To determine how sensitive each of the ADH1 models are, we performed LHS-PRCC sensitivity analysis. (A) Density scatter plots from LHS sampling of the parameter space show how the fit to data (y-axis) changes as a function of each parameter (x-axis). Correlations of the data in (A) are shown together for comparison in (B). The NO model is far less sensitive to variation in biological parameters. (TIF) [file pbio.1001528.s008.tiff]
