# Supplementary material for: Promoter Sequence Determines the Relationship between Expression Level and Noise
Source: PLoS Biol. 2013 Apr 2;11(4):e1001528. doi: 10.1371/journal.pbio.1001528 (PMC3614515; doi:10.1371/journal.pbio.1001528)
Supplement: Text S1 — This file contains supporting information. (DOCX) [file pbio.1001528.s013.docx]

# Supplemental information

Table of Contents

Supplemental information 1

Flow cytometry with a dual-reporter dual-gene system can accurately measure promoter-specific changes in intrinsic noise 1

Flow cytometry with an Elowitz style dual-reporter measures intrinsic noise similar to our dual-gene dual-reporter system 2

Sensitivity analysis 4

Robustness analysis 4

Parameter values of best fits 8

Supplementary Table 1. Cloned promoter sequences lengths 10

References 11

## Flow cytometry with a dual-reporter dual-gene system can accurately measure promoter-specific changes in intrinsic noise

It is important to note that quantification of changes in burst size and burst frequency through changes in noise assumes an intrinsic measurement of expression in which variability in expression between cells is a result of stochasticity at the promoter and not at the general transcriptional/translational machinery. Any extrinsic influences will distort the noise-burst frequency and noise strength-burst size relationships [1]. In order to correct for ‘extrinsic noise’ (expression capacity) [2] we used a dual-gene system with the promoter of interest driving YFP and a constitutively expressed mCherry, and use the ratio between YFP and mCherry as the measured expression level. Because mCherry expression does not change with zinc concentration (**Figure S1**), this normalization can be used to remove significant amounts of extrinsic variability consistently throughout induction. To further eliminate extrinsic variation we perform stringent gating on forward scatter, side scatter, and mCherry expression, which results in the removal of ~70-90% of the cell population. Finally, we note that we quantify changes in noise, not absolute noise, and therefore we only need to accurately measure changes in intrinsic noise, and not intrinsic noise itself. For these reasons we therefore expect that the reported YFP/mCherry measurements accurately reflect a combination of pathway specific and promoter specific noise. Furthermore, we believe that pathway specific noise represents a small fraction of the reported noise, and that this does not affect our results. The strongest evidence is that the noise for all promoters does not change uniformly with zinc or with measured ZAP1 promoter driven YFP. For example, noise of ADH1 and ADH3 is high at low zinc concentrations, while noise of ZRT2 and ZRT1 is low at low zinc concentrations. Furthermore, our ability to measure differences in noise between different promoter and ATG context mutants (which, presumably, do not affect pathway-specific noise), strongly suggests that intrinsic noise is the dominant source of noise in our measurements.

## Flow cytometry with an Elowitz style dual-reporter measures intrinsic noise similar to our dual-gene dual-reporter system

To validate our claim that the dual-gene dual-reporter system measures intrinsic noise, we constructed an Elowitz style dual-reporter for the ZRT2 promoter. To construct this strain we amplified by PCR the URA3-ZRT2_pr_-YFP-ADH1_term_ construct using primers with long tails to direct recombination into the his3 locus. We then transformed this PCR product into BY4741 and confirmed integration by PCR and sequencing. In addition we PCR amplified an mCherry-ADH1_term_-cloNAT^R^ construct using primers with identical tails, and inserted this PCR product by recombination into the his3 locus of BY4742. We then PCR amplified the URA3-ZRT2_pr_ construct by PCR using primers to direct homologous recombination in the newly created *his3*::mCherry-ADH1_term_ locus of BY4742. This resulted in *his3*::URA3-ZRT2_pr_-mCherry-ADH1_term_ MAT alpha strain and a *his3*::URA3-ZRT2_pr_-ADH1term MAT A strain, which we crossed and selected for diploids on SC-Met-Lys media. Figure 4d (inset) shows that intrinsic noise as a function of induction gives the characteristic curve that we observed using our dual-gene system.

This type of dual-reporter system is only valid if the distributions of the two fluorescent reporters are identical. When dealing with hundreds of events the Kolmogorov-Smirnov test can be used to compare two distributions [3]. However, for thousands of events obtained with flow cytometry the KS test is oversensitive [4] and gives meaningless results. In order to compare the YFP and mCherry expression distributions in the *ZRT2* promoter dual-reporter system we bootstrap downsampled the expression distributions to 500 cells. We find that at zinc concentrations less than 600uM the distributions of YFP and mCherry are identical (Figure S11). However, at higher zinc concentrations (lower Zap1 activity) mCherry has a wider distribution that YFP. Part of this non-equivalence is due to autofluorescence. Because maximal *ZRT2* induction occurs at ~100uM the difference in YFP and mCherry distributions does not affect our conclusion that intrinsic noise remains constant throughout the repressive regime. It does, however, impede our ability to accurately measure intrinsic noise using this system in the regime of low activation (high zinc).

## Sensitivity analysis

To investigate the robustness and sensitivity of the parameters of our model we perform a rigorous sensitivity analysis procedure described by [5]. First, we sample 10,000 instances of the model, each with a unique parameter setting. This sampling is done using a Latin Hypercube, to ensure uniform sampling in the multidimensional parameter space of our model. Next we do a rank transformation on the sampled instances using the distance of the model instances to the measured data (for both mean expression and noise) and quantify the correlation that each parameter has with the goodness of fit measure. Fig. S8 shows the scatter plots and correlation coefficient for each parameter of the ZRT1, ADH1 NO/TD and ZRT2 models.

We note that the NO model is significantly less sensitive than the TD model. The most sensitive parameter in the model is the scaling parameter. This parameter converts measured fluorescence to number of proteins and therefor we would expect this parameter to influence model outcome hugely.

## Robustness analysis

Barkai et al. describe a method of model validation that is based on the robustness of a model to parameter perturbations [6]. The idea is that a free parameter model will often have a parameter setting that fits the data, regardless of it being the right model. However, biological systems are noisy and parameter values are not fixed but fluctuate around some mean. These fluctuations do not prevent the biology from working. Therefore a model of a biological system should be robust to parameter perturbations. A model that is not robust is likely not the right model.

We proposed two different models for ADH1 repression. We found that although the NO model gave a better fit, both models have good fits to the measured data. To investigate which of the models is more robust we performed a perturbation analysis. For each of 100 best fits, and for each parameter, we sampled 10.000 perturbations in the range of -2 to 2 fold change. We did this for both alternative models. Fig. 3E shows the cumulative distribution of the goodness of fit (distance of model to data) for both models. We find that the NO model is significantly more robust than the TD model and therefore more likely to be the correct model for ADH1 repression.

**Notation and choice of parameter values**

While we do not know the precise values of many of the parameters in our model, biologically realistic bounds can be estimated for most parameters. We chose to set the upper and lower bounds of each free parameter as loose as reasonable, and we obtained good fits to data for a wide range of parameters. This robustness to parameter variation shows that the promoter-switching model accurately replicates the experimental data despite both uncertainties in the values of individual biochemical values and in experimental error.

Here follows a description of all parameters used in the model and their respective value constraints:

(All rate parameters are in minute^-1^)

1. **Protein degradation ( *δ* ).** YFP is highly stable. We therefore assume that protein degradation comes only from dilution. While the population doubling time does increase with as the zinc concentration drops below 150μM, per-cell rates of protein production also decrease with decreasing growth rate. For reasons of simplicity we chose to fix δ. This approximation does not affect our conclusions. We set the degradation rate *(δ)* to ln(2)/90 = 0.0077. Where 90 is the average measured doubling time (in minutes) in our experiments.
2. **Burst size ( *b* ).** Burst size is the average number of YFP molecules produced from each mRNA molecule. This parameter combines both translation and mRNA degradation assuming geometrically distributed protein abundance. Lu *et al.* [7] found that ~80% of yeast proteins have between 100-10,000 proteins per mRNA. We therefore set the bounds for expression of the yeast codon-optimized YFP to be 100 – 10,000_proteins/mRNA_.
3. **Transcription rate ( *r1 , r2, r3, r4* ).** The transcription rate is the rate of production of stable mRNAs while the promoter is in each respective state. Experimentally measured transcription rates combine both on and off promoter states. Therefore, the total rate of transcription for a gene is the sum, for all states, of the fraction of time spent in that state times the transcription rate in that state. The upper bound for expression rate is between 4_transcripts/minute_ [8] and 10 _transcripts/minute_ [9]. We note that these rates represent the combination of on and off promoter states. We set the transcription rate bounds to be 0-1 for off states, and 2-8 for on states.
4. **Promoter switching rates ( *Kon , Koff* ).** To estimate the minimum rate with which a promoter can switch between off and on we used expression data from the repressed *GAL1* promoter. We assume that this promoter is off most of the time, but occasionally switches on. Cai *et al.* measured a burst frequency of 0.2 events per cell-cycle for the *GAL1* promoter on a plasmid with a copy number of 10-40 [10]. We therefore set the minimum switching rate (*K_min_, see Eq.11,14,15*) to be 10^-4^ (0.2_transcripts/cell-cycle_ / 90_minutes/cell-cycle_ / 20_plasmid-copies/cell_). High *Kon* values for a TF means that the TF is essentially always bound, while high *Koff* values mean that the TF is never bound. We know that the off-switching rate for a promoter can be relatively rapid, as transcription of *POL1* occurs as a series of uncorrelated events, each resulting in the production of a single mRNA molecule. This implies that the promoter off switching rate can be high enough so that only a single transcription event occurs each time the promoter switches on. We therefore set the maximal switching rate (*K_max,_*) to be 10.
5. **Promoter switching as a function of [zinc].** We describe promoter switching with a hill equation as a function of [zinc]. This function has four parameters: K_min_ and K_max_ (described above), and [Zn]_mid_ and H, which is the [zinc] of half maximal K value (threshold) and Hill-coefficient (sensitivity) respectively. We allow [Zn]_mid_ to take on any value that is within the input [zinc] range – between 1 and 3000 uM zinc. H can be any positive value, where zero is a linear response and any non-zero positive value a sigmoidal response.
6. **Scaling of YFP to proteins/cells ( *S* ).** To convert measured fluorescence into molecules of YFP we determined how the measured YFP/mCherry ratio for each promoter at 3mM zinc compares to measured protein molecules / cell [11]. We find a normalized fluorescence to protein scaling of: YFP/mCherry * 0.0146 = Protein (molecules/cell). We allow the scaling to be a free parameter, within a range of 10 fold up and down from the determined scaling value.

## Parameter values of best fits

**ZRT1 (ATG):**

| *Kmin* | 10^-5.5014 |
| --- | --- |
| *Kmax* | 10^-0.9406 |
| *ZNmid* | 10^3.8179 |
| *H* | 10^0.5778 |
| *Koff* | 10^-0.7332 |
| *r1* | 10^0.0867 |
| *r2* | 10^-0.3211 |
| *b1 (each ATG variant has its own b)* | 550 |
| *b2* | 843 |
| *b3* | 1100 |
| *b4* | 904 |
| *b5* | 1430 |
| *b6* | 1312 |

**ADH1 NO:**

| *Kmin* | 10^-3.3292 |
| --- | --- |
| *Kmax* | 10^-0.8158 |
| *ZNmid* | 10^1.7836 |
| *H* | 10^2.2554 |
| *Koff* | 10^-0.6999 |
| *r1* | 10^0.0331 |
| *r2* | 10^2.0000 |
| *b* | 10^2.7879 |

**ADH1 TD:**

| *Kmin* | 10^-2.8635 |
| --- | --- |
| *Kmax* | 10^-0.5724 |
| *ZNmid* | 10^1.9606 |
| *H* | 10^3.2449 |
| *Kon* | 10^-1.8496 |
| *r1* | 10^0 |
| *r2* | 10^2 |
| *b* | 10^2.4002 |

**ZRT2:**

| *Kmin* | 10^-6.0000 |
| --- | --- |
| *Kmax* | 10^5.3546 |
| *ZNmid* | 10^2.0913 |
| *H* | 10^0.1965 |
| *S Kon_rep (ratio Kon_act to Kon_rep)* | 10^1.2548 |
| *Koff_act* | 10^-0.4506 |
| *S Koff_rep (ratio Koff_act to Koff_rep)* | 10^-0.1878 |
| *r1* | 10^0.3281 |
| *r2* | 10^5.0000 |
| *r3* | 10^0 |
| *r4* | 10^0.2273 |
| *b* | 10^3.1694 |

## References

1. Friedman N, Cai L, Xie X (2006) Linking Stochastic Dynamics to Population Distribution: An Analytical Framework of Gene Expression. Phys Rev Lett 97. doi:10.1103/PhysRevLett.97.168302.

2. Colman-Lerner A, Gordon A, Serra E, Chin T, Resnekov O, et al. (2005) Regulated cell-to-cell variation in a cell-fate decision system. Nature 437: 699–706. doi:10.1038/nature03998.

3. Young IT (1977) Proof without prejudice: use of the Kolmogorov-Smirnov test for the analysis of histograms from flow systems and other sources. J Histochem Cytochem 25: 935–941.

4. Finch PD (1979) Substantive difference and the analysis of histograms from very large samples. J Histochem Cytochem 27: 800.

5. Marino S, Hogue IB, Ray CJ, Kirschner DE (2008) A methodology for performing global uncertainty and sensitivity analysis in systems biology. Journal of Theoretical Biology 254: 178–196. doi:10.1016/j.jtbi.2008.04.0

6. Barkai N, Leibler S (1997) Robustness in simple biochemical networks. Nature 387: 913–917. doi:10.1038/43199.

7. Lu P, Vogel C, Wang R, Yao X, Marcotte EM (2007) Absolute protein expression profiling estimates the relative contributions of transcriptional and translational regulation. Nature Biotechnology 25: 117–124. doi:10.1038/nbt1270.

8. Pelechano V, Chávez S, Pérez-Ortín JE (2010) A complete set of nascent transcription rates for yeast genes. PLoS ONE 5: e15442. doi:10.1371/journal.pone.0015442.

9. Iyer V, Struhl K (1996) Absolute mRNA levels and transcriptional initiation rates in Saccharomyces cerevisiae. Proc Natl Acad Sci USA 93: 5208–5212.

10. Cai L, Friedman N, Xie XS (2006) Stochastic protein expression in individual cells at the single molecule level. Nature 440: 358–362. doi:10.1038/nature04599.

11. Wang M, Weiss M, Simonovic M, Haertinger G, Schrimpf SP, et al. (2012) PaxDb, a database of protein abundance averages across all three domains of life. Mol Cell Proteomics. doi:10.1074/mcp.O111.014704.
